# Potential therapeutic effects and pharmacological evidence of sinomenine in central nervous system disorders

**DOI:** 10.3389/fphar.2022.1015035

**Published:** 2022-09-16

**Authors:** Hongxiang Hong, Xu Lu, Qun Lu, Chao Huang, Zhiming Cui

**Affiliations:** ^1^ Department of Spine Surgery, The Second Affiliated Hospital of Nantong University, Nantong, Jiangsu, China; ^2^ Department of Pharmacology, School of Pharmacy, Nantong University, Nantong, Jiangsu, China; ^3^ Department of Pharmacy, Nantong Third Hospital Affiliated to Nantong University, Nantong, Jiangsu, China

**Keywords:** sinomenine, oxidative stress, neuroinflammation, autophagy, apoptosis

## Abstract

Sinomenine is a natural compound extracted from the medicinal plant Sinomenium acutum. Its supplementation has been shown to present benefits in a variety of animal models of central nervous system (CNS) disorders, such as cerebral ischemia, intracerebral hemorrhage, traumatic brain injury (TBI), Alzheimer’s disease (AD), Parkinson’s disease (PD), epilepsy, depression, multiple sclerosis, morphine tolerance, and glioma. Therefore, sinomenine is now considered a potential agent for the prevention and/or treatment of CNS disorders. Mechanistic studies have shown that inhibition of oxidative stress, microglia- or astrocyte-mediated neuroinflammation, and neuronal apoptosis are common mechanisms for the neuroprotective effects of sinomenine. Other mechanisms, including activation of nuclear factor E2-related factor 2 (*Nrf2*), induction of autophagy in response to inhibition of protein kinase B (Akt)-mammalian target of rapamycin (mTOR), and activation of cyclic adenosine monophosphate-response element-binding protein (CREB) and brain-derived neurotrophic factor (BDNF), may also mediate the anti-glioma and neuroprotective effects of sinomenine. Sinomenine treatment has also been shown to enhance dopamine receptor D2 (DRD2)-mediated nuclear translocation of αB-crystallin (CRYAB) in astrocytes, thereby suppressing neuroinflammation *via* inhibition of Signal Transducer and Activator of Transcription 3 (STAT3). In addition, sinomenine supplementation can suppress N-methyl-D-aspartate (NMDA) receptor-mediated Ca^2+^ influx and induce γ-aminobutyric acid type A (GABA_A_) receptor-mediated Cl^−^ influx, each of which contributes to the improvement of morphine dependence and sleep disturbance. In this review, we outline the pharmacological effects and possible mechanisms of sinomenine in CNS disorders to advance the development of sinomenine as a new drug for the treatment of CNS disorders.

## Introduction

**FIGURE 1:**
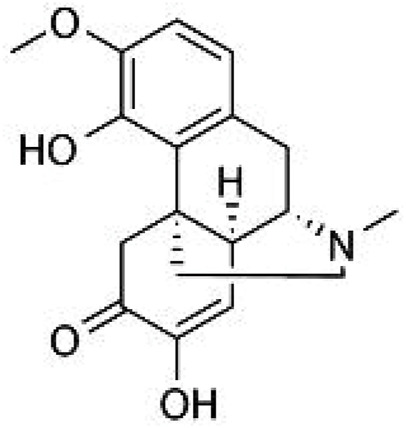
The structure of sinomenine.

Sinomenine (Figure 1; molecular formula: C_19_H_23_NO_4_; molecular weight: 329.39; solvent: dimethyl sulfoxide) is a natural compound extracted from the medicinal plant Sinomenium acutum. Its pharmacodynamic properties have been described in numerous studies. Researchers have found that the oral bioavailability of sinomenine in rats is about 80% and that sinomenine has a protein binding rate of more than 60% when administered orally. Once distributed in the body, sinomenine can be metabolized in the liver and excreted rapidly *via* the kidneys (Tsai and Wu, 2003; Liu et al., 2005; Gao et al., 2019a). At high doses, administration of sinomenine can cause a number of side effects, such as edema and itching, convulsive central excitation, vomiting and defecation, and vasodilatation and reddening, most of which could be reduced by classical antihistamines (Fu et al., 1963; Yamasaki, 1976; Gao et al., 2019a). However, it is worth noting that repeated administration *via* the oral route of administration has been shown not to result in over-accumulation of sinomenine in the body, including the brain (Liu et al., 1996; Gao T. et al., 2019a), suggesting that oral administration may be a suitable route of administration for the clinical use of sinomenine. Indeed, this hypothesis was supported by the previous use of sinomenine in healthy male volunteers (Yan et al., 1997).

Pharmacological studies have shown that sinomenine has therapeutic effects in various diseases, including rheumatoid arthritis (Xu et al., 2008), pain (Jiang et al., 2020), atherosclerosis (Zhang et al., 2021), and cancer (Gao et al., 2019b). These effects of sinomenine are mainly due to its multi-factorial pharmacological actions, such as anti-inflammation, anti-oxidative stress, and anti-neoplasm (Xu et al., 2008; Gao et al., 2019b; Jiang et al., 2020; Zhang et al., 2021). In addition to its effects on peripheral tissues and organs, sinomenine also shows apparent benefits in a variety of animal models of central nervous system (CNS) disorders due to its ability to rapidly penetrate the brain (Liu et al., 2005; Long et al., 2010; Gao et al., 2019a). Therefore, taking sinomenine has been reported to be beneficial in preventing or ameliorating a number of CNS disorders, including cerebral ischemia, intracerebral hemorrhage, traumatic brain injury (TBI), Alzheimer’s disease (AD), Parkinson’s disease (PD), epilepsy, depression, multiple sclerosis, morphine tolerance, and glioma. Sinomenine is now considered a potential agent for the prevention and/or treatment of CNS disorders. However, to date, the outlined understandings about the role of sinomenine in CNS disorders remain unclear.

In this review, we addressed this issue by summarizing and discussing the pharmacological effects of sinomenine and its possible mechanisms in the prevention and/or therapy of CNS disorders based on an online literature search. Because the regulatory effects and possible mechanisms of sinomenine in pain have been well studied and discussed in previous studies (Gao et al., 2013; Zhu et al., 2016; Gao et al., 2019b; Jiang et al., 2020; Gao et al., 2021), the pharmacological aspects of sinomenine in pain were not included in this review. We hope this review will advance the use of sinomenine and sinomenine-containing products in CNS disorders.

### Pharmacoligcal effect of sinomenine in cerebral ischemia

Cerebral ischemia is a global disease that threatens people around the world and can lead to inability to work or live and even high mortality rates. It usually occurs in conditions such as stroke and hypo-perfusion, especially in patients with other cardiovascular factors such as hyperlipaemia (Alloubani et al., 2021) and obesity (Ganna and Anna, 2022). Therapy for cerebral ischemia is still a difficult issue in the clinic. Most therapies only alleviate the disease process but cannot fully restore brain function, which is associated with significant economic and social burdens. Therefore, the search for new drugs to treat the damage caused by cerebral ischemia is a hot topic for researchers and drug manufacturers.

In a previous study, intraperitoneal administration of sinomenine at a dose of 10 or 20 mg/kg (once daily, 3 days after surgery, administered 30 min or 6 h after surgery) was shown to attenuate cerebral infarction, cerebral edema, neuronal apoptosis, and neurological deficits caused by middle cerebral artery occlusion (MCAO) in mice (Qiu et al., 2016a; Qiu et al., 2016b) (Table 1). Administration of sinomenine by tail vein injection (90 mg/kg) 1 h before ischemia can reduce the neurological severity, infarct volume, brain water content, and the deficient of blood-brain barrier permeability caused by MCAO in rats (Yang et al., 2016a) (Table 1). Pretreatment with sinomenine (30 mg/kg, i.p., 12 h before MCAO and again 0.5 h before the start of reperfusion after 120 min of MCAO) can also reduce cerebral infarcts in rats (Wu et al., 2011) (Table 1). In addition, 20 mg/kg sinomenine injected intraperitoneally into mice (3 days) after MCAO was found to inhibit the MCAO-induced decrease in neuron numbers and increase in brain water content in mice (Bi et al., 2021) (Table 1). These results suggest that sinomenine may be a potential drug for the treatment of cerebral ischemic injury.

**TABLE 1:** Comprehensive information about the pharmacological effects and mechanisms of sinomenine in models of cerebral ischemia.

Pharmacological effect	Object	Drug administration	Possible mechanisms	References
*Reduce cerebral infarction, cerebral edema, and neuronal apoptosis	Mice	*10 or 20 mg/kg	*Suppress *NLRP3 complex activation* and pro-inflammatory cytokine expression	Qiu et al. (2016a), Qiu et al. (2016b)
		*i.p.		
*Improve neurological deficits		*Once daily, 3 days after surgery	*Inhibit astrocyte activation, STAT3 phosphorylation, and CRYAB expression in astrocytes	
*Prevent neurological severity, infarct volume, and brain water content	Rats	*90 mg/kg	*Prevent lactic acid and lactic dehydrogenase increase	Shen Yang et al. (2016)
		*Tail vein injection		
*Restore blood-brain barrier permeability		*1 h before ischemia	*Reduce Bax/Bcl-2 ratio *inhibit ASIC1a-Ca^2+^-CaMKII signaling	
Reduce cerebral infarcts	Rats	*30 mg/kg	Reduce cerebral infarcts	Wu et al. (2011)
		*i.p.		
		*12 h before MCAO and again 0.5 h before reperfusion after 120 min of MCAO		
Suppress OGD/R-induced PC12 cell death	Cultured rat cortical neurons	0.1, 0.5, 1, or 5 μM, 24 h before OGD until the end of recovery	*Inhibit KCl-induced intracellular Ca^2+^ increase	Wu et al. (2011)
			*Inhibit ASIC1a- and voltage-gated L-type calcium channel-mediated currents	
*Inhibit neuron decrease	Mice	*20 mg/kg	Increase Nrf-2-mediated antioxidant response	Bi et al. (2021)
*reduce brain water content		*i.p.		
		*3 days after MCAO		
*suppress OGD-induced pro-inflammatory cytokine expression	*Cultured mixed glia * BV-2 microglia	*0.1, 0.5, or 1.0 mM, 12 h	Suppress NLRP3 complex activation	Qiu et al. (2016a)
		*25, 50, or 100 μM		
		*50, 100, or 200 μM, 2–24 h		Bi et al. (2021)
Prevent OGD-induced pro-inflammatory cytokine production	BV-2 microglia	25, 50, or 100 μM, pretreated for 2 h	*Reduce SP1/miRNA-183-5p expression	Qin et al. (2018)
			*Increase IκB-α expression	
			*Reduce NF-κB activation	

The neuroprotective effects of sinomenine in cerebral ischemia are mediated by several mechanisms. The first consideration is inhibition of microglia-mediated neuroinflammation. In animal models of cerebral ischemia, sinomenine can reduce nucleotide-binding oligomerization domain-like receptor thermal protein domain-associated protein 3 (*NLRP3*), apoptosis-associated speck-like protein containing caspase recruitment domain (ASC), cleaved caspase-1, interleukin-1β (IL-1β), IL-6, tumor necrosis factor-α (TNF-α), and IL-18 expression in ischemic hemispheric tissues in animals with MCAO (Qiu et al., 2016a) (Table 1). *In vitro* studies showed that incubation with sinomenine (0.1, 0.5, or 1.0 mM, 12 h; 25, 50, or 100 μM; or 50, 100, or 200 μM, 2–24 h) suppressed the oxygen-glucose deprivation (OGD)-induced increase in NLRP3, cleaved caspase-1, TNF-α, IL-6, IL-1β, or inducible nitric oxide synthase (iNOS) expression in cultured mixed glia or BV-2 microglia (Qiu et al., 2016a; Bi et al., 2021) (Table 1).

**FIGURE 2:**
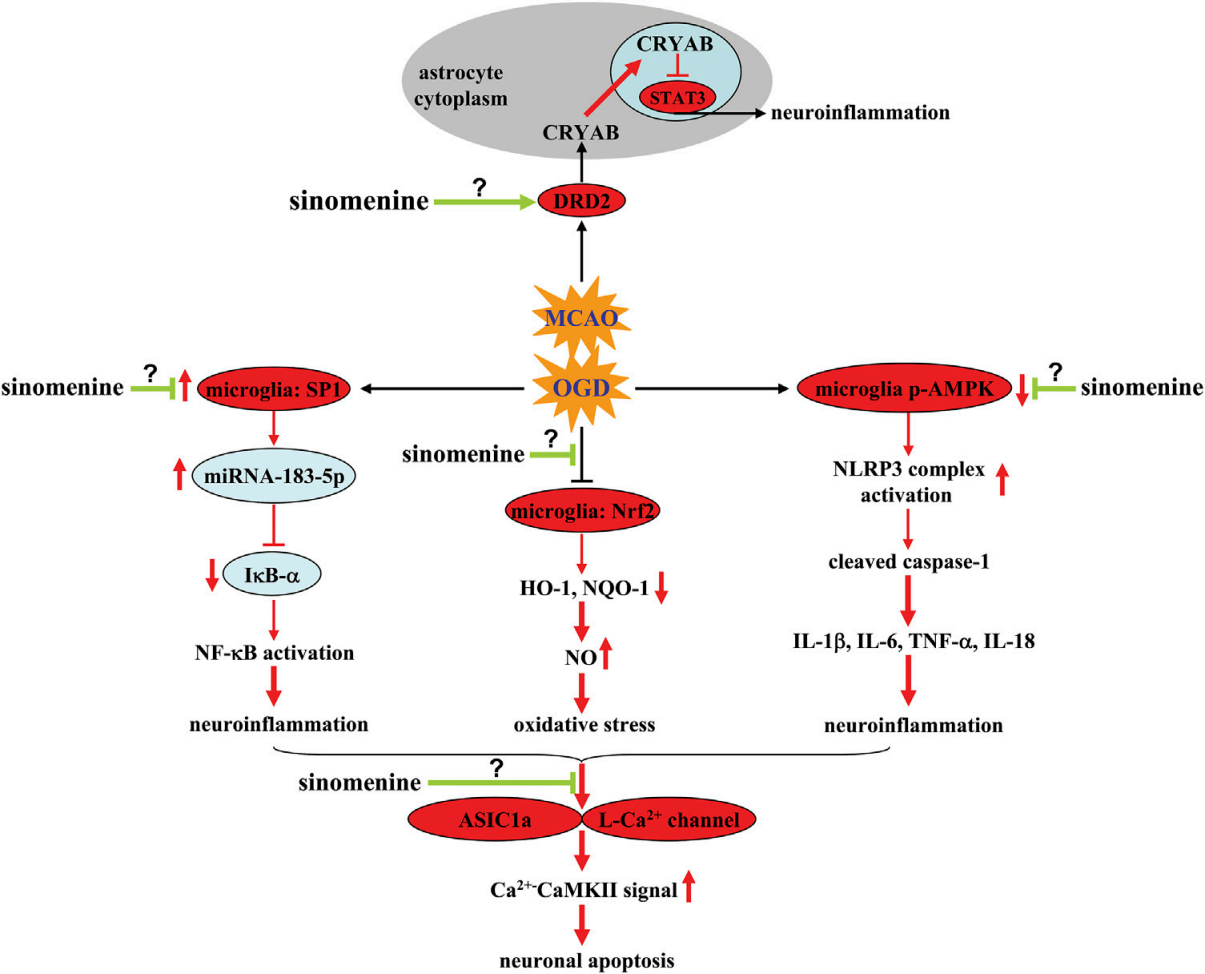
Effects and mechanisms of sinomenine in cerebral ischemia. Sinomenine can suppress the OGD-induced increase in SP1 and miRNA-183-5p expression in microglia, thereby causing the decrease in IκB-α expression by targeting the 3′-UTR site of mouse IκB-α, which subsequently reduces NF-κB activation and neuroinflammation Qin et al. (2018). Sinomenine may also suppress NO-mediated oxidative stress by restoring impaired Nrf2 and subsequent HO-1-NQO1 signaling in microglia Bi et al. (2021). Moreover, sinomenine can reverse OGD-induced dephosphorylation of AMPK in microglia, resulting in inhibition of the NLRP3 complex, which in turn reduces the overproduction of pro-inflammatory cytokines Qiu et al. (2016a). Sinomenine can also enhance DRD2-mediated CRYAB nuclear translocation and subsequent suppression of nuclear STAT3 and neuroinflammation in MCAO-stimulated brain tissues and OGD-stimulated astrocytes Qiu et al. (2016b). Inhibition of ASIC1a- or L-type Ca^2+^ channels-mediated Ca^2+^-CaMKII signaling is also a potential mechanism for the anti-apoptotic effect of sinomenine in neurons Wu et al. (2011), Yang et al. (2016a). However, it is currently unclear how sinomenine suppresses neuronal ASIC1a and L-type Ca^2+^ channels, suppresses microglial SP1, and increases phospho-AMPK in microglia or DRD2 function in astrocytes.

Mechanistic studies showed that the inhibitory effect of sinomenine on neuroinflammation in microglia was mediated by suppression of OGD/R-induced phosphorylation of nuclear factor-κB (NF-κB) and down-regulation of inhibitor of κB-α (IκB-α) in BV-2 microglia (Qin et al., 2018) (Figure 2; Table 1). The effect of sinomenine on IκB-α may be related to its down-regulatory effect on the expression levels of miRNA-183-5p, a miRNA with a target site in the 3′-UTR of mouse IκB-α that is thought to negatively correlate with IκB-α expression. Transcription factor specificity protein 1 (*SP1*) can bind to miRNA-183-5p *via* the transcription factor binding site (TFBS) and positively regulate miRNA-183-5p expression (Figure 2; Table 1) (Qin et al., 2018). Incubation with sinomenine can prevent the OGD-induced increase of *SP1* in BV-2 microglia, thereby increasing the expression of miRNA-183-5p and inducing the decrease of IκB-α, which subsequently induces NF-κB activation and the production of pro-inflammatory cytokines (Figure 2; Table 1) (Qin et al., 2018). The inhibitory effect of sinomenine on neuroinflammation under *in vitro* conditions could also be mediated by adenosine 5′-monophosphate-activated protein kinase (AMPK): Incubation with sinomenine can increase the phosphorylation levels of AMPK in OGD-stimulated mixed glial cells, and inhibition of AMPK abrogates the sinomenine-induced decrease in NLRP3, cleaved caspase-1, and IL-1β expression in OGD-stimulated mixed glial cells (Figure 2; Table 1) (Qiu et al., 2016a). However, before confirming the role of SP1 and AMPK in the anti-neuroinflammatory effects of sinomenine under ischemic conditions, investigators should investigate how sinomenine inhibits SP1 and activates AMPK.

The neuroprotective effect of sinomenine in cerebral ischemia might also be related to its inhibitory effect on astrocyte-mediated inflammatory responses. Administration of sinomenine was found to inhibit astrocyte activation and the phosphorylation of Signal Transducer and Activator of Transcription 3 (STAT3) and increase the expression of αB-crystallin (CRYAB) in ischemic brain tissue after MCAO (Figure 2) (Qiu et al., 2016b). *In vitro* studies showed that the above effects of sinomenine occurred mainly in astrocytes, as incubation with sinomenine (1 mM, 24 h) can block OGD-induced activation of STAT3 and generation of pro-inflammatory cytokines in cultured astrocytes, which was abrogated by knocking down CRYAB (Figure 2; Table 1) (Qiu et al., 2016b). Moreover, incubation with sinomenine (1 mM, 24 h) can induce the up-regulation and nuclear translocation of CRYAB in primary cultured astrocytes and enhance the interaction between CRYAB and STAT3, which in turn inhibits the DNA-binding activity of STAT3 (Figure 2; Table 1) (Qiu et al., 2016b). Therefore, sinomenine may produce an anti-inflammatory effect in astrocytes by promoting the translocation of CRYAB into the nuclear, thereby enhancing the targeting of CRYAB to inhibit STAT3.

Although CRYAB can mediate the anti-inflammatory effects of sinomenine in astrocytes, it remains unclear how sinomenine enhances CRYAB signaling in astrocytes. Astrocytic dopamine receptor D2 (DRD2), which has been reported to suppress neuroinflammation (Zhu et al., 2018), is a molecule upstream of CRYAB (Shao et al., 2013). The anti-inflammatory effect induced by the activation of astrocytic DRD2 in the CNS can be blocked by suppressing CRYAB (Qiu et al., 2016b). Administration of sinomenine can increase the expression of DRD2 in both ischemic brain tissue after MCAO and cultured astrocytes stimulated by OGD (Qiu et al., 2016b) (Table 1). Moreover, the suppressive effect of sinomenine incubation on OGD-induced STAT3 activation and pro-inflammatory response in cultured astrocytes can be abolished by knocking down DRD2 (Figure 2; Table 1) (Qiu et al., 2016b). Therefore, it is speculated that DRD2 mediates the enhancing effect of sinomenine on CRYAB expression in OGD astrocytes, thereby suppressing the binding of STAT3 to the promoter of proinflammatory cytokine genes (Figure 2). And sinomenine may be a potential DRD2 agonist. However, how sinomenine increases DRD2 expression under ischemia conditions in astrocytes remains unclear.

In addition to neuroinflammation, oxidative stress is another important pathological process that mediates the pathogenesis of cerebral ischemia (Higashi et al., 2021). The neuroinflammatory response is linked to oxidative stress *via* iNOS, which produces nitric oxide (NO) (Madrigal et al., 2002; Yang et al., 2017). On the one hand, administration of sinomenine was found to increase nuclear translocation of NF-E2-related factor 2 (*Nrf2*) and expression of heme oxygenase-*1* (*HO-1*) *and* nicotinamide adenine dinucleotide phosphate quinine oxidoreductase 1 (NQO1) in ischemic brain tissue in MCAO animals (Bi et al., 2021) (Table 1). Incubation with sinomenine (50, 100, or 200 μM, 2–24 h) also induces Nrf2 nuclear accumulation and increase HO-1 and NQO1 mRNA in BV-2 microglia, which was accompanied by a significant decrease in iNOS expression (Figure 2; Table 1) (Bi et al., 2021). Decreased iNOS expression leads to a decrease in NO, thereby reducing oxido-nitrosative stress and mediating the neuroprotective effects of sinomenine in cerebral ischemia. On the other hand, high levels of oxido-nitrosative stress may promote the progression of neuroinflammation. Knockdown of the Nrf2 gene has been shown to abolish the inhibitory effect of sinomenine on the expression of pro-inflammatory mediators and enhance the effect of sinomenine on anti-inflammatory mediators such as IL-10 and arginase-1 in BV-2 microglia (Figure 2; Table 1) (Bi et al., 2021). Therefore, the neuroprotective effects of sinomenine might also be mediated by Nrf2-mediated anti-neuroinflammatory response.

One of the major cellular consequences of cerebral ischemia is neuronal apoptosis when stimulated by neuroinflammation or oxido-nitrosative stress. Previous studies have shown that administration of sinomenine can prevent the MCAO-induced increase in lactic acid and lactic dehydrogenase in the ischemic brain tissue of MCAO rats (Yang et al., 2016a). In addition, pretreatment with sinomenine can increase the expression of caspase-3 in ischemic brain tissue of MCAO rats. *In vitro* studies showed that pre-incubation with sinomenine (0.1, 0.5, 1, or 5 μM, 24 h before OGD until the end of recovery) can prevent OGD/R-induced PC12 cell death (Wu et al., 2011) (Table 1). Neuronal apoptosis triggered by neuroinflammation and oxidative stress may be mediated by several mechanisms, such as intracellular Ca^2+^ overload, decrease in anti-apoptosis proteins, and increase in pro-apoptosis proteins. It was shown that sinomenine administration reduced the expression of acid-sensing ion channel 1a (ASIC1a) and the expression of Ca^2+^-coupling phospho-calmodulin-dependent protein kinase II (CaMKII), which subsequently reversed the MCAO-induced increase Bax/Bcl-2 ratio in ischemic brain tissue (Figure 2; Table 1) (Yang et al., 2016a). Furthermore, *in vitro* studies showed that extracellular incubation with sinomenine could inhibit ASIC1a- and voltage-gated L-type calcium channel-mediated currents and KCl-induced increase in intracellular Ca^2+^ in cultured rat cortical neurons (Figure 2; Table 1) (Wu et al., 2011). These results suggest that inhibition of intracellular Ca^2+^ overload mediated by ASICs or L-type voltage-gated calcium channels may be an important mechanism for the neuroprotective effect of sinomenine in cerebral ischemia. However, the exact mechanism for the inhibition of ASICs or L-type voltage-gated calcium channels by sinomenine remains to be determined. Investigators should clarify whether sinomenine can bind directly to these channels. Researchers should also investigate whether the regulatory effect of sinomenine on neuroinflammation and oxidative stress is related to its regulatory effect on ASICs or L-type voltage-gated calcium channels.

### Pharmacological effects of sinomenine in intracerebral hemorrhage

Subarachnoid hemorrhage (SAH) is a serious CNS disease associated with a high mortality rate. During the pathogenesis of SAH, the infiltration of blood into the subarachnoid space of the brain can reduce cerebral blood flow and trigger the activation and infiltration of immune cells, which subsequently leads to impaired body functions (Xu et al., 2021; Chen et al., 2022a). Therefore, inhibition of neuroinflammation may be a potential strategy for the treatment of SAH. Previous studies have reported that administration of sinomenine (i.p., 20 mg/kg, once daily, 3 days) can suppress intracerebral hemorrhage (ICH)-induced brain edema and neurologic damage in mice (Shi et al., 2016) (Table 2). This effect of sinomenine may be related to anti-neuroinflammation because sinomenine can prevent neuron death and apoptosis induced by conditioned medium of cultured microglia treated with erythrocyte lysate by inhibiting caspase-3 (Yang et al., 2014) (Table 2). Further analysis showed that pre-incubation with sinomenine (0.1 or 1 mM) prevented erythrocyte lysate (3 days)-induced microglia migration and IL-1β, TNF-α, and iNOS expression in primary cultured microglia, while it increased the expression of anti-inflammatory mediators (IL-10 and arginase-1) in microglia stimulated by erythrocyte lysate (Yang et al., 2014) (Table 2). This suggests that sinomenine attenuates ICH-induced neurological impairment, probably by shifting microglia toward an anti-inflammatory phenotype. This hypothesis is supported by another *in vivo* study which found that sinomenine suppressed ICH-induced microglial infiltration and iNOS, IL-1β, TNF-α, and matrix metalloproteinase-3/9 (MMP-3/9) expression through NF-κB inhibition, while increasing the expression of IL-10 and arginase-1 in damaged brain tissue from ICH mice (Shi et al., 2016) (Table 2).

**TABLE 2:** Comprehensive information about the pharmacological effects and mechanisms of sinomenine in models of intracerebral hemorrhage, traumatic brain injury, Alzheimer’s disease, Parkinson’s disease, disorders associated with neuronal hyper-activation, depression, multiple sclerosis, and morphine dependence.

Pharmacological effect	Object	Drug administration	Possible mechanisms	References
Intracerebral hemorrhage				
Suppress brain edema and neurologic damage	Mice	*20 mg/kg	Shift microglia to an anti-inflammatory phenotype	Shi et al. (2016)
		*i.p.		
		*once daily, 3 days		
Prevent neuronal death and apoptosis induced by conditioned medium from microglia treated with erythrocyte lysate	*Neuron; *Microglia	*0.1 or 1 mM, *60 min before erythrocyte lysate simulation	Shift microglia to an anti-inflammatory phenotype	Yang et al. (2014)
Traumatic brain injury				
*Suppress neurological deficits and brain water increase	Mice	*30 or 70 mg/kg	Increase Nrf-2-mediated antioxidant response	Fu et al. (2018)
		*i.p.		
*Suppress neuronal apoptosis		*24 h		
*Alleviate cerebral edema and neuronal apoptosis	Mice	*10, 30, or 50 mg/kg	Increase Nrf-2-mediated antioxidant response	Youqing Yang et al. (2016)
		*i.p.		
*Improve motor performance		*24 h		
*Attenuate neuroinflammation	Rabbits	*10, 30, or 50 mg/kg	Shift microglia to an anti-inflammatory phenotype	Sharma et al. (2020)
		*i.p.		
		*30 min after surgery, 1 day		
Alzheimer’s disease				
Prevent cell death induced by conditioned medium from oligomeric Aβ-treated astrocytes	*HT22 cells	*100 μM *1.5 h before stimuli	Prevent pro-inflamamtory mediator production	Shukla and Sharma (2011), Singh et al. (2020)
	*Cultured hippocampal neurons			
	*C8D1A cells			
	*Cultured human astrocytes			
	*BV-2 microglia			
Reverse trimethyltin-induced 1) increase in discrimination index in novel object detection, 2) impairment of alternation in the short-term Y maze, 3) decrease in step-through latency in the passive avoidance paradigm, and 4) increase in probe trial error and latency in the Barnes maze task in rats	Rats	*100 mg/kg	*Increase Nrf-2-mediated antioxidant response	Rostami et al. (2022)
		*p.o.	*Suppress AChE activity	
		*1 h after stimuli, once daily, 3 weeks	*Suppress BACE1 activity	
Parkinson’s disease				
*Suppress MPTP-induced motor impairment, *increase *TH*-positive neurons	Mice	*20 mg/kg	Enhance autophagy by inhibiting the Akt-mTOR signaling	Bao et al. (2022)
		*i.p.		
		*5 days before MPTP treatment and another 4 days for a total of 9 days		
*Prevent LPS- or MPP^+^-induced impairment of dopamine take up *prevent LPS-induced decrease in TH-positive neurons	Midbrain neuron-enriched cultures	10^−6^, 10^−5^, 10^−14^, or 10^−13^ M	Inhibit iNOS expression and TNF-α, PGE2, and NO production	Qian et al. (2007)
Disorders associated with neuronal hyper-activation				
*Prevent kainate-induced status epilepticus, *prevent kainate-induced hippocampal DNA fragmentation and neuronal reduction	Rats	*50 mg/kg	*Enhance antioxidant response	Ramazi et al. (2020)
		*p.o.		
		*once daily, started 4 days before till day 3 after kainate injection	*Inhibit neuroinflammation	
Suppress pentylenetetrazole-induced decrease in seizure latency and duration	Rats	*20, 40, or 80 mg/kg	*Inhibit NLRP1-inflammasome complex activation and neuroinflammation	Gao et al. (2018)
		*i.p.		
		*once daily, 29 days		
*Shorten sleep latency	*Mice	*40 mg/kg	*Promote Cl- flux	Yoo et al. (2017)
		*p.o.		
*Prolong total sleep time	*Hypothalamic neurons	*administered 60 min before behavioral tests	*Increase GABA_A_ receptor and GAD65/67 expression	
Depression				
Reverse CUS-induced depression-like behaviors	Mice	*30, 100 or 300 mg/kg	*Reverse NLRP3-inflammasome complex activation	Liu et al. (2018)
		*p.o.		
		*once daily, 21 days	*Reverse p38 and NF-κB activation	
Reverse CSDS-induced depression-like behaviors	Mice	*20 or 40 mg/kg	Restore the BDNF-CREB signaling	Li et al. (2018)
		*i.p.		
		* once daily, 14 days		
Multiple sclerosis				
Reduce neurological scores associated with clinical symptoms of multiple sclerosis	Mice	*100 mg/kg	Suppress neuroinflammation	Kiasalari et al. (2021)
		*i.p.		
		*once daily, 18 or 19 days		
*Prevent weight loss	Mice	*50, 100, or 200 mg/kg	Suppress neuroinflammation	Zeng et al. (2007)
		*i.p.		
*Delay disease progression associated with EAE		*once daily, 5 days		
Reduce EAE scores	Mice	*15 mg/kg	Suppress neuroinflammation	Yan et al. (2010)
		*i.p.		
		*treated from day 1–40 after MOG_35-55_ immunization		
Morphine dependence				
Prevent morphine-induced increase in time spent in the non-preferred white compartment in the conditioned place preference test	Mice	*80 mg/kg	*Reduce TH and NR2B expression	Fang et al. (2017)
		*i.p.		
		*on days 5–7 after the preconditioning phase and the first and second sessions on day 4	*Increase MOR expression	
Prevent morphine-induced conditioned place preference	Mice	*60 mg/kg	*Inhibit morphine-induced activation of astrocytes	Ou et al. (2018)
		*i.p.		
		*45 min before morphine injection, 3 days		
Reverse morphine-induced 1) increase in Fusobacteria and decrease in Actinobacteria, 2) decrease in tight junction proteins and OPRM1 and OPRD1, and 3) increase in levels of DRD2A, HTR2A, BDNF, and NTRK2 in the zebrafish brain and/or intestine	Zebrafish	80 mg/kg	Regulate the homeostasis of gut microbiota	Chen et al. (2020)

The next important question that should be answered is how sinomenine suppresses NF-κB activation under ICH conditions: inhibition of NF-κB nuclear translocation, NF-κB transcriptional activity, or only NF-κB expression? CRYAB expression triggered by DRD2 activation is a potential way to limit neuroinflammatory responses in the brain by inhibiting NF-κB nuclear translocation (Zhang et al., 2015; Huang et al., 2020; Yang et al., 2022). Since sinomenine can enhance the expression of DRD2 and its downstream molecule CRYAB in astrocytes and ischemic brain tissue (Qiu et al., 2016b), it is reasonable to speculate that sinomenine may restrict NF-κB activation in microglia by mobilizing DRD2-mediated CRYAB expression in the pathological process of ICH. However, this is currently only a hypothesis. We do not yet know the cellular basis for the regulation of neuroinflammation by DRD2-mediated CRYAB expression in animal models of ICH. These questions should be carefully investigated in future studies.

### Pharmacological effects of sinomenine in traumatic brain injury

Traumatic brain injury (TBI) is a common CNS disorder that causes both rapid and delayed damage and results in high morbidity and mortality, but for which there are currently no effective treatments (Sabet et al., 2021). Therefore, it is necessary to develop new strategies for the treatment of TBI. Previous studies have reported that intraperitoneal administration of sinomenine (i.p., 30 or 70 mg/kg; 10, 30, or 50 mg/kg) 30 min after TBI can suppress neurological deficits and brain water content in mice, accompanied by a significant decrease in neuron death and apoptosis (Yang et al., 2016b; Fu et al., 2018) (Table 2). This finding is also evidenced by the decrease in caspase-3 expression, the decrease in mitochondrial Bax and cytoplasmic Bcl-2 and cytochrome C expression, and the increase in cytoplasmic Bax and mitochondrial Bcl-2 and cytochrome C expression in the ipsilateral cortex (Fu et al., 2018) (Table 2). These results indicate that sinomenine exerts neuroprotective effects in animal models of TBI, probably by suppressing Bax translocation to mitochondria and cytochrome C release into the cytoplasm.

Further studies showed that administration of sinomenine (10, 30, or 50 mg/kg, i.p., 30 min after surgery, 1 day) in TBI mice can reduce the TBI-induced increase in malondialdehyde (MDA) and decrease in mitochondrial GPx and superoxide dismutase (SOD) activities in the ipsilateral cortex by promoting the translocation of Nrf2 into the nucleus (Yang et al., 2016b) (Table 2). Researchers have also found that administration of D-sinomenine conjugate (intravenous; 400 μl, 30 mg/kg; administered 4 h after injury), a modified sinomenine conjugated to hydroxyl terminated generation-4 poly dendrimer that has rapid cellular uptake ability and specifically accumulates in microglia in injured brain areas, attenuates pro-inflammatory cytokine expression in injured brain areas in TBI kits by shifting microglia to an anti-inflammatory phenotype (Sharma et al., 2020) (Table 2). The above results suggest that sinomenine properly protects neurons in injured brains from TBI by inhibiting oxidative stress and neuroinflammation. Future studies should investigate how sinomenine suppresses neuroinflammation and increases Nrf2 activation in the injured brains of animals with TBI.

### Pharmacological effects of sinomenine in Alzheimer’s disease

**FIGURE 3:**
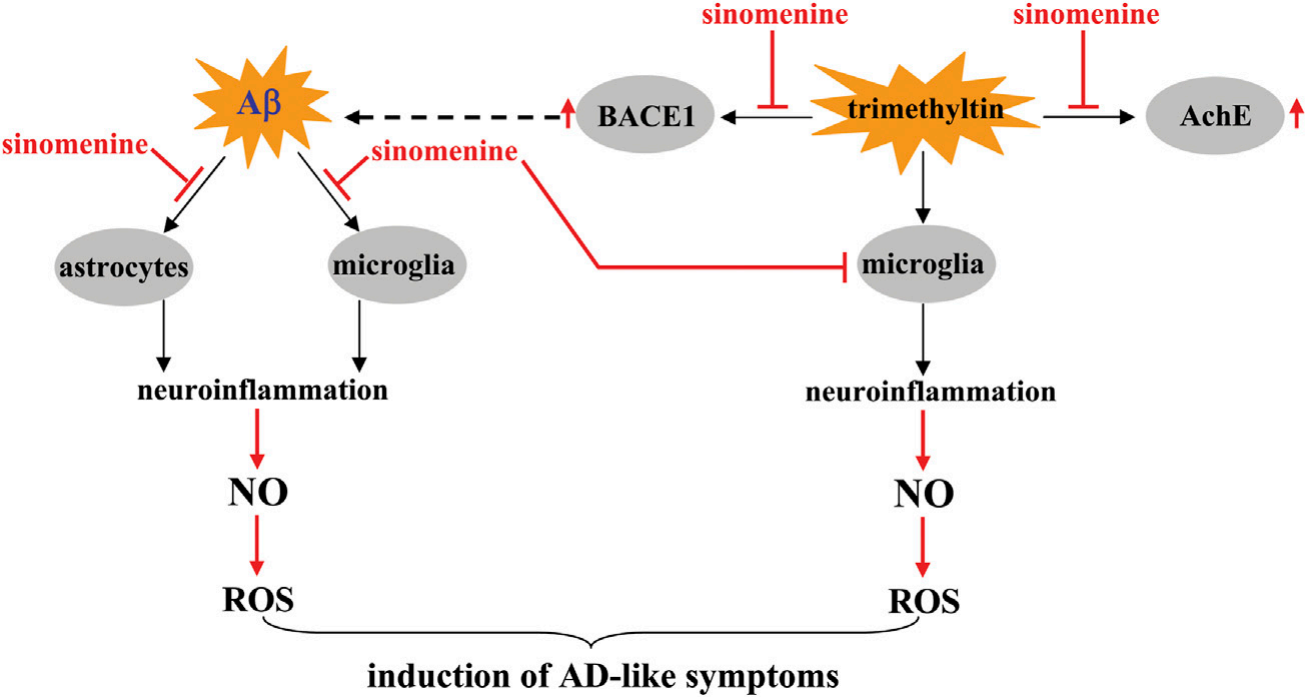
Effects and mechanisms of sinomenine in pathological conditions similar to AD. Sinomenine can suppress Aβ-triggered neuroinflammation mediated by microglia and astrocytes, thereby reducing the accumulation of NO, which in turn reduces the production of ROS Shukla and Sharma (2011), Singh et al. (2020). Under trimethyltin-stimulated conditions, sinomenine suppresses the progression of neuroinflammation mediated by microglia, thereby also reducing the accumulation of NO and ROS Rostami et al. (2022). Meanwhile, sinomenine was found to suppress the trimethyltin-induced overexpression of AchE and BACE1 in brain tissue and reduce the accumulation of a pathological trait protein Aβ in the brain through the latter effect Rostami et al. (2022).

AD is a common neurodegenerative disorder associated with progressive deterioration of cognitive abilities, learning skills, and memory and imposes a severe economic and social burden. In the clinic, there are no effective drugs to cure this frightening disease, although many hypotheses such as the Aβ- and tau-accumulation hypotheses have been raised (Dai et al., 2022; Roda et al., 2022). Therefore, it is necessary to search for new drugs to treat AD. Previous studies have reported that pre-incubation with sinomenine (100 μM, 1.5 h before stimuli) can inhibit oligomeric amyloid β (Aβ; 100 μM, 24 h)-induced production of monocyte chemoattractant protein-1 (MCP-1) and NO, IL-6, IL-18, IL-1β, TNF-α, and interferon-γ (IFN-γ) in mouse C8D1A astrocytic cell lines and primary cultured human astrocytes (Figure 3; Table 2), accompanied by a significant decrease in cell death in cultured HT22 cells and primary cultured hippocampal neurons induced by conditioned medium from oligomeric Aβ-treated astrocytes (Singh et al., 2020). It was also found that pre-incubation with sinomenine (100 μM) 1.5 h before and during pathological stimuli counteracted the increase in levels of NO, IL-6, TNF-α, and MCP-1 induced by oligomeric Aβ (100 μM, 12 h) in BV2 microglia and protected HT22 hippocampal cells and primary hippocampal neurons from indirect toxicity mediated by Aβ-treated microglia (Shukla and Sharma, 2011) (Table 2). In addition, incubation with sinomenine can suppress the overproduction of ROS induced by Aβ in mouse C8D1A astrocytic cells, cultured human astrocytes, and BV-2 microglia (Figure 3; Table 2) (Shukla and Sharma, 2011; Singh et al., 2020). Considering that NO can promote the formation of ROS, the above results suggest that inhibition of neuroinflammation and subsequent oxidative stress may contribute to the anti-AD effect of sinomenine. However, it is worth noting that the above studies also found that Aβ incubation triggered a significant increase in IL-10 levels in astrocytes that was prevented by sinomenine pre-incubation (Singh et al., 2020). This result seems to contradict most previously published studies in which IL-10 was decreased under Aβ stimulation conditions (Morgese et al., 2021; Ren et al., 2022). Future studies should be performed to elucidate the exact reasons for this phenomenon.

Although the anti-AD effect of sinomenine has so far been inferred only from *in vitro* studies, a previous study has shown that sinomenine may be able to alleviate the disease symptoms of AD. Administration of sinomenine (p.o.; 1 h after the stimuli, once daily, 3 weeks, 100 mg/kg) reverses the trimethyltin-induced 1) increase in discrimination index in novel object detection, 2) impairment of alternation in the short-term Y maze, 3) decrease in step-through latency in the passive avoidance paradigm, and 4) increase in probe trial error and latency in the Barnes maze task in rats (Rostami et al., 2022) (Table 2). Further analysis revealed that sinomenine administration could suppress the trimethyltin-induced increase in MDA, ROS, NO, TNF-α, and IL-6 and the trimethyltin-induced decrease in SOD in rat hippocampal tissue (Figure 3; Table 2) (Rostami et al., 2022). These results support the conclusions from the *in vitro* studies and suggest that suppression of neuroinflammation and oxidative stress is critical for the effect of sinomenine against AD. Curiously, sinomenine did not affect the levels of *glutathione* (GSH), catalase, glutathione reductase, and glutathione peroxidase in the hippocampus of trimethyltin-treated rats (Rostami et al., 2022) (Table 2), suggesting that the effect of sinomenine against oxidative stress in this trimethyltin model may not be comparable to the effect observed in other models of CNS disorders. Together with the previously reported effect of sinomenine on IL-10 in Aβ-stimulated astrocytes (Singh et al., 2020) and the fact that IL-10 is a classical anti-neuroinflammatory factor, researchers should investigate why sinomenine selectively affects the processes involving pro-neuroinflammatory responses and pro-oxidative stress.

Acetylcholinesterase (AChE) is a molecule that has important functions in AD. AchE promotes brain dysfunction by degrading acetylcholine (Sivaraman et al., 2022). Inhibition of AchE expression or activity is a potential strategy for the treatment of AD. Administration of sinomenine was shown to suppress the trimethyltin-induced increase in AChE activity in rat hippocampal tissue (Figure 3; Table 2) (Rostami et al., 2022), suggesting that inhibition of AchE is a potential mechanism for the anti-AD effect of sinomenine. In addition to AchE, β-secretase 1 (BACE1) is another molecule that may mediate the pathogenesis of AD. It is responsible for the proteolytic processing of the amyloid precursor molecule and the formation of Aβ fragments (Taylor et al., 2022). Administration of sinomenine can suppress the increase in BACE1 activity induced by trimethyltin in rat hippocampal tissue (Figure 3; Table 2), suggesting that inhibition of BACE1 may be also a possible mechanism for the anti-AD effect of sinomenine (Rostami et al., 2022). Because Aβ accumulation may be an important pathogenesis for AD, clarifying whether sinomenine administration could reduce Aβ accumulation in the brain could help confirm the anti-AD effect of sinomenine. Since the trimethyltin-induced learning and memory impairment is not a classic model for AD, researchers should investigate whether sinomenine indeed has an effect against AD using different animal models for AD.

### Pharmacological effects of sinomenine in Parkinson’s disease

PD is a common neurodegenerative disorder characterized by progressive degeneration of dopaminergic neurons in the substantia nigra pars compacta and subsequent depletion of an important neurotransmitter in the striatum that can negatively control motor skills—dopamine (Nemade et al., 2021). Numerous therapeutic methods are available in the clinic, including drug and non-drug treatments, to treat PD. However, considering the low efficacy of treatment and severe side effects, the development of new drugs is always a hot topic for PD research. Administration of sinomenine (i.p., 20 mg/kg, 5 days before treatment with 1-methyl-4-phenyl-1,2,3,6-tetrahydropyridine (MPTP) and for another 4 days) can suppress the MPTP-induced decline in rotarod time, pounds for grip strength, and forepaw stride length in mice, which was accompanied by an increase in tyrosine hydroxylase (*TH*)-positive cells in the substantia nigra pars compacta (Bao et al., 2022) (Table 2), suggesting that sinomenine administration improves motor skills in mice with PD by promoting the survival of dopaminergic neurons.

**FIGURE 4:**
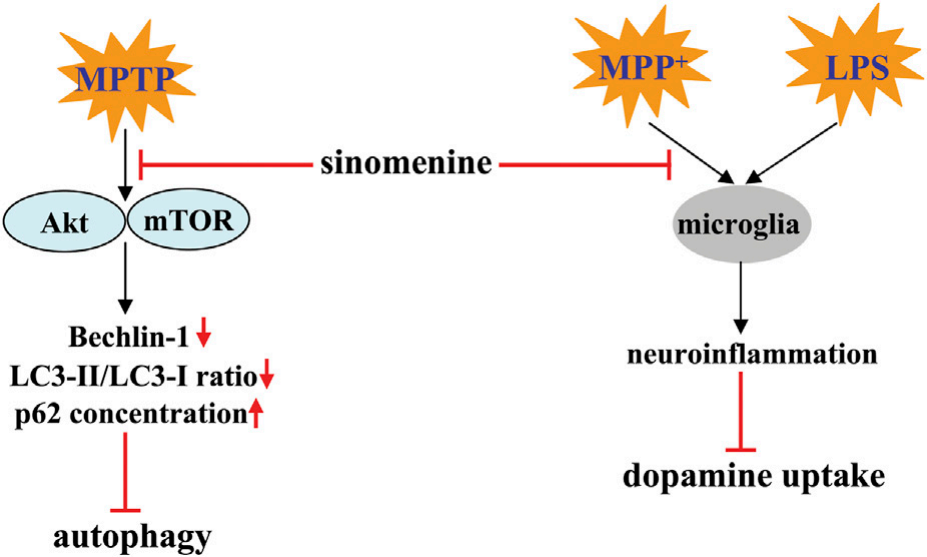
Effects and mechanisms of sinomenine in pathological conditions resembling PD. Sinomenine can inhibit MPTP-induced dephosphorylation of Akt and mTOR, thereby promoting the process of autophagy by increasing the expression of Beclin-1, increasing the LC3-II/LC3-I ratio, and reducing the accumulation of p62 Bao et al. (2022). Sinomenine may also suppress neuroinflammation triggered by MPP^+^ or LPS, thereby increasing dopamine uptake Qian et al. (2007).

Autophagy is a general biological process that mediates the pathogenesis of PD (Sepúlveda et al., 2022). Promoting autophagy is considered a potential strategy for the treatment of PD. Administration of sinomenine can suppress the MPTP-induced decrease in Beclin-1 and LC3-II/LC3-I ratio, as well as the MPTP-induced increase in p62 in the substantia nigra pars compacta of the mouse brain, by inhibiting the protein kinase B (Akt)-mammalian target of rapamycin (mTOR) signaling (Figure 4; Table 2) (Bao et al., 2022), suggesting that triggering autophagy may mediate the action of sinomenine against PD. This hypothesis is supported by the finding that MHY1485, a specific activator of the Akt-mTOR signaling pathway that affects autophagy, further impairs the motor abilities of PD mice and attenuates the beneficial effect of sinomenine on the motor abilities of PD mice by promoting the loss of dopaminergic neurons (Bao et al., 2022).

Another mechanism for the anti-PD effect of sinomenine may be related to the inhibition of neuroinflammation, because pre-incubation with sinomenine (10^−6^, 10^−5^, 10^−14^, 10^−13^ M) can simultaneously prevent the LPS-induced decrease in the ability of midbrain neuron-enriched cultures to take up dopamine and the LPS-induced decrease in TH-positive neurons in midbrain neuron-enriched cultures (Figure 4; Table 2) (Qian et al., 2007). Mechanistic studies showed that the neuroprotective effect of sinomenine in the above model may be highly related to the attenuating effect of sinomenine on the LPS-induced production of TNF-α, prostaglandin E2 (PGE2), and NO, as well as on the expression of iNOS in rat microglia-enriched cultures (Figure 4; Table 2) (Qian et al., 2007). This hypothesis is also supported by the finding that incubation with sinomenine prevented the MPP^+^-induced decrease in dopamine uptake in primary mesencephalic neuron-glia cultures by microglia: Sinomenine showed no effect on the toxic effects of 1-methyl-4-phenylpyridiniumion (MPP^+^) in primary mesencephalic neuron cultures, which was restored when the neuron-enriched cultures were reconstituted with purified microglia but not astrocytes (Qian et al., 2007) (Table 2). Thus, it is possible that sinomenine does not directly protect neurons from MPP^+^-mediated toxicity, but that targeting microglia is a crucial process. However, it is worth noting that although microglia are known to mediate the pathogenesis of PD through neuroinflammation, sinomenine did not suppress MPP^+^-induced production of TNF-α and NO in the presence of microglia, but suppressed MPP^+^-induced production of superoxide in rat mesencephalic neuron-glia cultures, suggesting that inhibition of superoxide-associated oxidative stress, but not neuroinflammation, may mediate the anti-neurotoxic effect of sinomenine (Qian et al., 2007).

### Pharmacological effects of sinomenine in disorders assocaited with neuronal hyperactivity

Epilepsy is a common disorder of the CNS characterized by an abnormal increase in neuronal excitability. Although there are a variety of drugs with different mechanisms of action for the treatment of epilepsy, 20%–30% of people are resistant to these drugs (Cardenas-Rodriguez et al., 2013). Therefore, finding new drugs to calm the over-activated neurons is an urgent goal for researchers. In a kainate model of temporal lobe epilepsy in rats, administration of sinomenine (50 mg/kg, once daily, p.o., started 4 days before to day 3 after kainate injection) prevented the severity of seizures and the onset of kainate-induced status epilepticus, which was associated with significant reversal of kainate-induced hippocampal aberrant MFS, DNA fragmentation, and neuronal reduction (Ramazi et al., 2020) (Table 2). In addition, administration of sinomenine (20, 40, or 80 mg/kg, i.p., once daily, 30 min before pentylenetetrazole injection, 29 days) was found to suppress the pentylenetetrazole-induced decrease in seizure latency and duration in rats, which was associated with a significant decrease spatial learning and memory impairment (Gao et al., 2018) (Table 2), suggesting that sinomenine is able to disrupt the kindling process.

**FIGURE 5:**
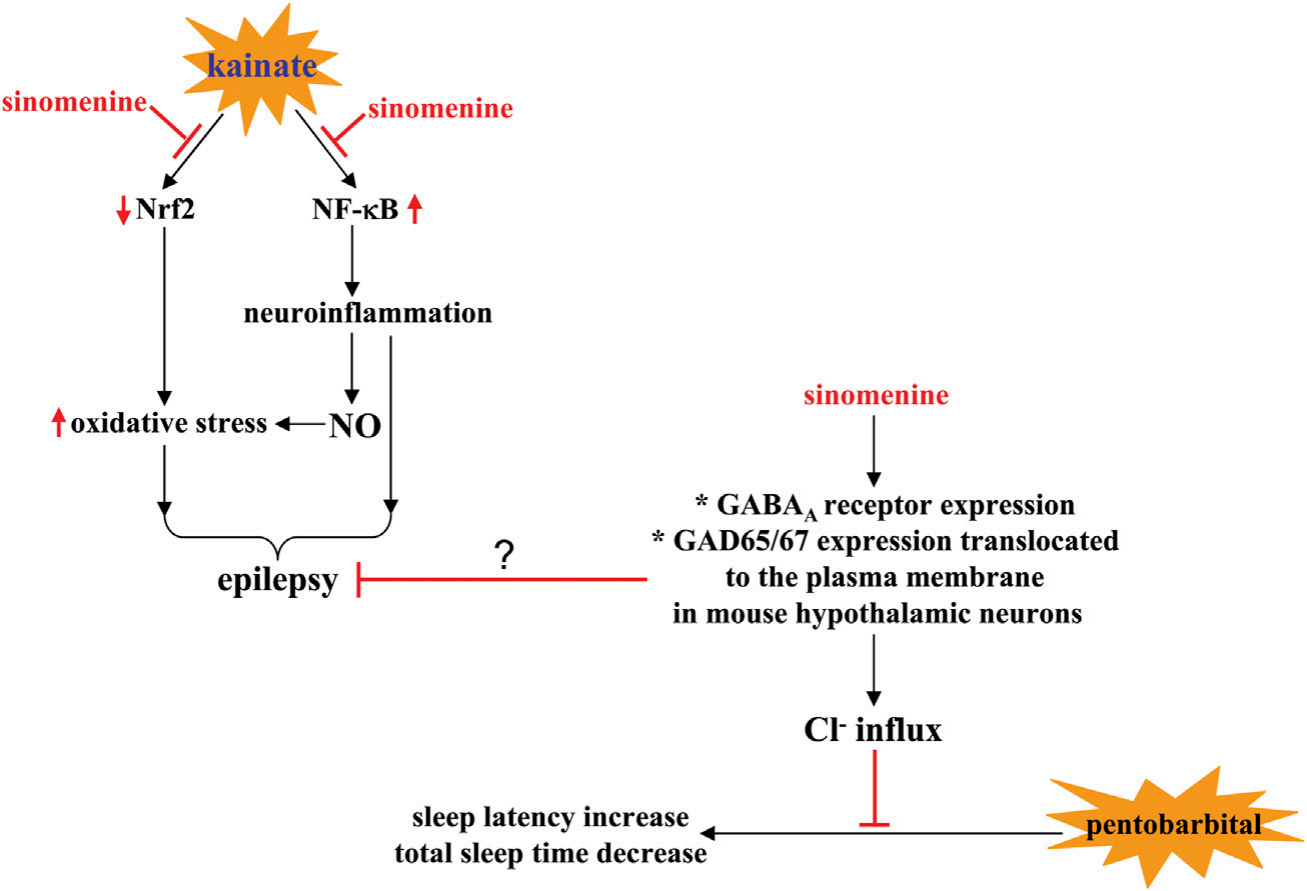
Effects and mechanisms of sinomenine in pathological conditions associated with neuronal hyperactivity. On the one hand, sinomenine administration can calm the pathological processes associated with epilepsy by suppressing kainate-induced impairment of Nrf2 signaling and the subsequent generation of oxidative stress or by suppressing NF-κB-mediated neuroinflammation and the production of NO in the brain Ramazi et al. (2020). On the other hand, sinomenine may increase sleep latency and shorten total sleep time in pentobarbital-treated animals by promoting Cl- flux, a process thought to be related to the increase in GABA_A_ receptor expression and GAD65/67 expression translocated to the plasma membrane in mouse hypothalamic neurons Yoo et al. (2017).

Researchers have also found that sinomenine administration can prevent the kainate-induced increase in hippocampal DNA fragmentation and caspase-1 levels and suppress the pentylenetetrazole-induced increase in Bax/Bcl-2 ratio in the rat hippocampus (Gao et al., 2018; Ramazi et al., 2020) (Table 2). Oxidative stress and neuroinflammation are two important factors that promote neuronal apoptosis in epilepsy (Parsons et al., 2022). The anti-epilepsy effect of sinomenine may be related to the changes in these two processes. On the one hand, sinomenine administration was found to suppress the kainate-induced increase in ROS and MDA levels and the kainate-induced decrease in HO-1 and SOD levels in the hippocampus (Figure 5) (Ramazi et al., 2020) (Table 2). On the other hand, prior administration of sinomenine prevented the kainate-induced increase in hippocampal NF-κB, toll-like receptor-4 (TLR-4), TNF-α, and glial fibrillary acidic protein (GFAP) levels in the hippocampus (Ramazi et al., 2020), as well as pentylenetetrazole-induced increases in the expression of NLRP1-inflammasome complexes such as NLRP1 and ASC, and pentylenetetrazole-induced increases in IL-1β, IL-18, IL-6, and TNF-α in rat hippocampus (Figure 5; Table 2) (Gao et al., 2018). Further studies should be performed to investigate whether the anti-oxidative stress and anti-neuroinflammatory effects of sinomenine are indeed linked in the pathogenesis of epilepsy. Another phenomenon that should be mentioned is that although sinomenine exhibits anti-oxidative stress effect in the above epilepsy model, no changes in hippocampal GSH levels were observed (Ramazi et al., 2020), which is very similar to the effect of sinomenine observed in the trimethyltin-induced model of AD (Rostami et al., 2022). Future studies should investigate why sinomenine selectively affects the processes associated with pro-oxidative stress in animal models of epilepsy.

Insomnia, a widespread phenomenon in modern society that can cause a variety of individual problems, such as increased anxiety and impaired ability to work, is another disease related to neuronal hyperactivity (Zeitzer, 2013; Carpena et al., 2021). Calming the over-activated neurons is a hopeful strategy for the treatment of disorders associated with sleep disturbance including insomnia. Previous studies have shown that administration of sinomenine (40 mg/kg, p.o., administered 60 min before behavioral tests) can shorten sleep latency and prolong total sleep time in mice treated with a hypnotic or sub-hypnotic dose of pentobarbital, suggesting that sinomenine may help to improve sleep disturbances (Figure 5; Table 2) (Yoo et al., 2017). However, because sinomenine administration can also reduce locomotor activity in mice (Yoo et al., 2017) and shows antidepressant effects in several animal models of depression (Li et al., 2018; Liu et al., 2018), it is necessary to further investigate the relationship between the sleep-inducing effect and the changes in spontaneous locomotor activity in mice.

It is known that Cl^−^ influx mediated by the γ-aminobutyric acid type A_A_ (GABA_A_) receptor is an important way for the brain to calm neuronal activity (Sieghart et al., 2022). The decrease in GABA_A_ receptor function may be an important mechanism for the development of neuronal hyperactivity. Previously published *in vitro* studies have shown that incubation with sinomenine (10 µM) increases intracellular Cl^−^ influx in primary cultured hypothalamic cells and subsequently causes neuronal hyperpolarization and appropriate inhibition of neuronal activity (Yoo et al., 2017) (Table 2). Administration of sinomenine (p.o., 40 mg/kg) can increase the expression of GABA_A_ receptors and the intensity of glutamate decarboxylase 65/67 (GAD65/67) expression translocated to the plasma membrane in mouse hypothalamic neurons (Figure 5; Table 2) (Yoo et al., 2017). These results indicate that the increased Cl^−^ influx in sinomenine-treated primary cultured hypothalamic cells may be due to the increase in GABA_A_ receptor functions (Figure 5). However, it is worth noting that in mouse hypothalamic neurons, only some (including the α4-, β1-, β2-, γ3-, but not the α5-subtype) but not all subtypes of GABA_A_ receptors are regulated by sinomenine (Yoo et al., 2017). This phenomenon should be given special attention because different subtypes of GABA_A_ receptors have different functions. Clarifying the reason for the selective regulation of GABA_A_ receptors by sinomenine could help unravel the broader functions of sinomenine in the CNS and could be particularly useful for developing drugs to regulate neuronal activities in various types of CNS disorders. Because the development of epilepsy is associated with an imbalance in GABA receptor function (Hernandez and Macdonald, 2019) and drugs aiming at improving GABA receptor function are used to treat epilepsy (Chuang and Reddy, 2018), future studies should investigate whether sinomenine suppresses the onset of epilepsy by improving GABA receptor function (Figure 5).

### Pharmacological effects of sinomenine in depression

Depression is a common CNS disorder that can be treated with conventional antidepressants developed according to the monoamine dysfunction hypothesis, such as the selective serotonin reuptake inhibitors (García-Marín et al., 2022). However, these drugs exhibit a number of problems, such as low response rate (Kruizinga et al., 2021), the occurrence of sexual dysfunction (Bakr et al., 2022), and increasing suicide rates (Braun et al., 2016). Therefore, it is necessary to develop new drugs for the treatment of depression. Previous studies have reported that administration of sinomenine (30, 100 or 300 mg/kg, p.o., once daily, 21 days; 20 or 40 mg/kg, i.p., 14 days, once daily) in mice can reverse the depression-like behaviors induced by chronic unpredictable stress (CUS)-induced in the sucrose preference test (SPT), forced swimming test (FST), and tail suspension test (TST) (Liu et al., 2018), as well as chronic social defeat stress (CSDS)-induced social interaction impairment in the social interaction test (SIT) and sucrose intake decline in the SPT (Li et al., 2018) (Table 2). In stress-naïve mice, it was also found that a single administration of sinomenine (20 and 40 mg/kg, i.p., 30 min before the behavioral test) shortened the immobility time in the FST and TST (Li et al., 2018). These results suggest that sinomenine may be a potential drug with antidepressant effects.

**FIGURE 6:**
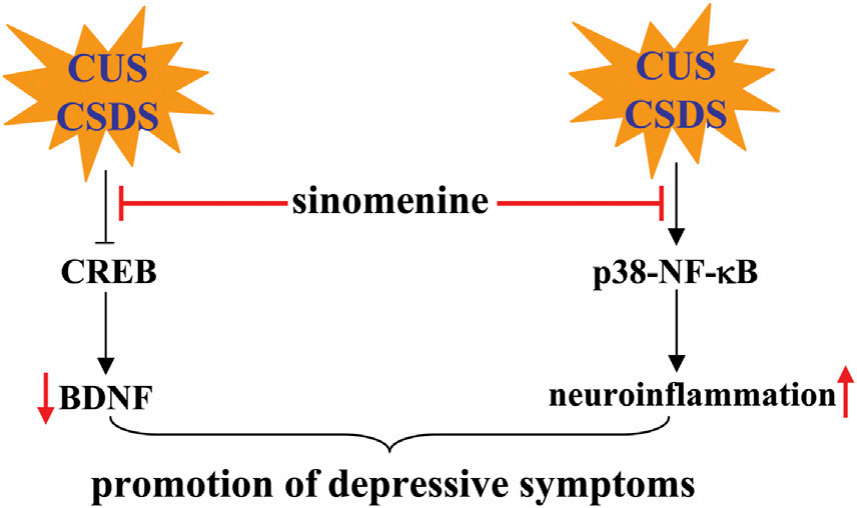
Effects and mechanisms of sinomenine in depression. Administration of sinomenine may ameliorate the pathogenesis of depression induced by CUS or CSDS by improving the function of BDNF-CREB signaling or reducing neuroinflammation mediated by p38 and NF-κB Li et al. (2018), Liu et al. (2018).

It is known that the development of depression is related to a variety of mechanisms, such as neuroinflammation (Yirmiya et al., 2015), brain-derived neurotrophic factor (BDNF) dysfunction (Poon et al., 2021), and monoamine dysfunction (García-Marín et al., 2022). Inhibition of neuroinflammation may mediate the antidepressant effect of sinomenine, because administration of sinomenine (30, 100, or 300 mg/kg; oral administration, 21 days) can suppress the CUS-induced expression of IL-1β, IL-6, TNF-α, and NLRP3-inflammasome complexes such as NLRP3, ASC, and caspase-1 in the hippocampus of mice, probably by inhibiting p38 and NF-κB (Figure 6; Table 2) (Liu et al., 2018). The enhanced neuroinflammatory response may mediate the pathogenesis of depression by triggering a variety of downstream pathogenesis processes, such as impairment of the monoamine system and cyclic adenosine monophosphate (cAMP)-response element binding protein (CREB)-BDNF signaling. Sinomenine administration can reverse the CSDS-induced decrease in phospho-CREB, phospho-tyrosine kinase receptor B (TrkB), and BDNF in the hippocampus and medial prefrontal cortex of mice (Figure 6; Table 2) (Li et al., 2018). Inhibition of TrkB signaling but serotonin function can reverse the reducing effect of sinomenine on immobility time in the FST and TST and the promoting effect of sinomenine on sucrose intake in stress-naïve mice, as well as the suppressing effect of sinomenine on CSDS-induced decrease in social interaction in the SIT (Li et al., 2018) (Table 2), suggesting that the antidepressant effects of sinomenine are mediated by BDNF rather than serotonin function. However, it is worth noting that another study by Liu et al. (2018) found that sinomenine administration reversed the CUS-induced decline in noradrenaline and serotonin levels in the hippocampus of mice, which seems to contradict another finding that serotonin depletion does not affect the antidepressant effects of sinomenine in mice (Li et al., 2018). At present, it is still unclear why the researchers observed different phenomena in the regulation of sinomenine on the serotonin system. One possible explanation could be the difference in experimental models.

### Pharmacological effects of sinomenine in multiple sclerosis

Multiple sclerosis is a well-known disease of the CNS with progressive demyelination and axonal damage and loss (Simkins et al., 2021). Although multiple sclerosis is a very active area of research, little is known about the causes and mechanisms underlying pathogenesis. Uncontrolled inflammation that produces high levels of pro-inflammatory factors is a key factor promoting the development of multiple sclerosis (Healy et al., 2022). Currently, the regulation of chronic inflammation and pathogenesis triggered by chronic stress are the focus of research.

**FIGURE 7:**
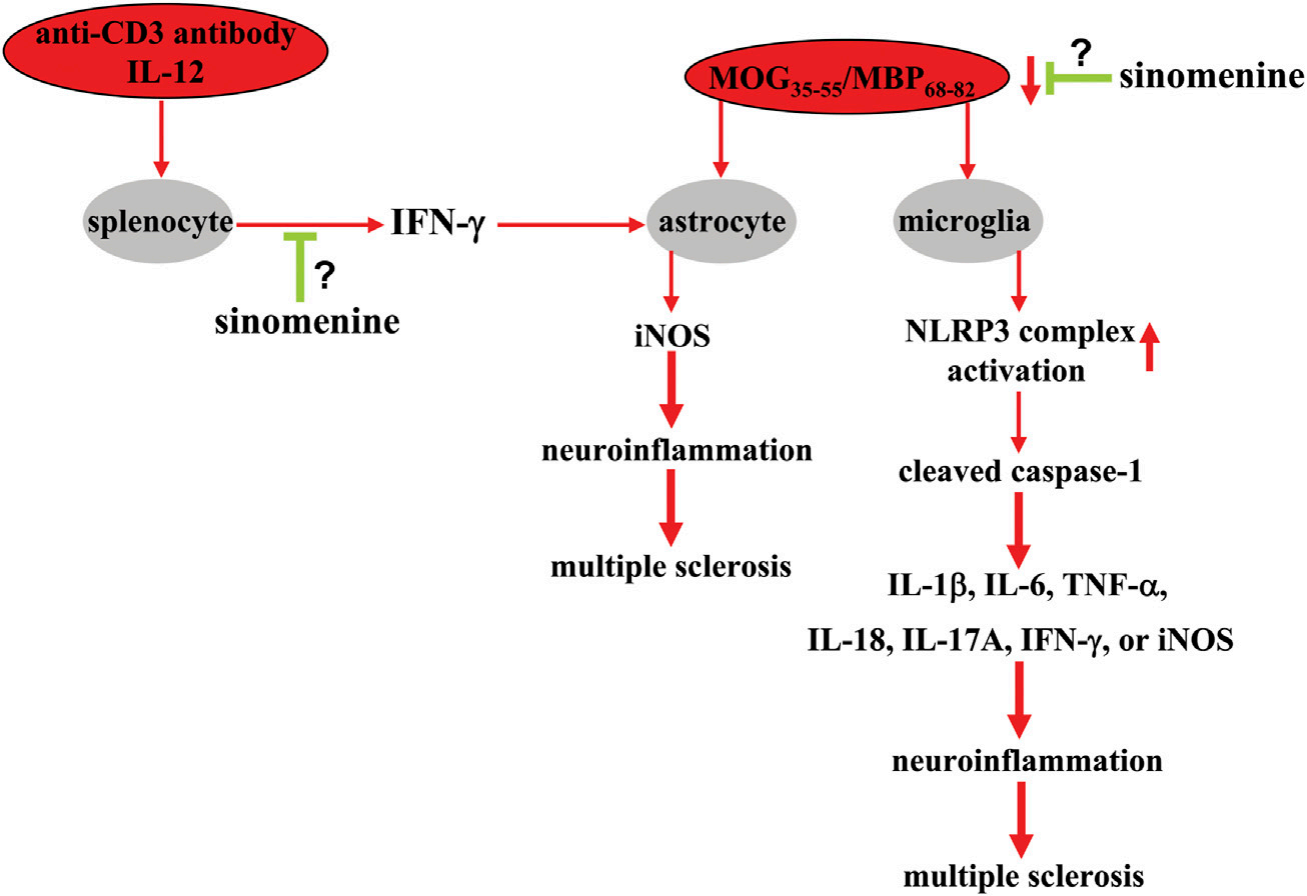
Effects and mechanisms of sinomenine in multiple sclerosis. Administration of sinomenine can suppress the pathological processes triggered by MOG_35-55_ or MBP_68-82_ in the context of multiple sclerosis by suppressing the progression of neuroinflammation in astrocytes and microglia by reducing the activation of the NLRP3 complex Zeng et al. (2007), Kiasalari et al. (2021). Sinomenine can also suppress the overproduction of iNOS in astrocytes triggered by a molecule derived from the anti-CD3 antibody and IL-12-mobilized splenocytes, INF-γ Gu et al. (2012). At present, it is unclear how sinomenine suppresses INF-γ production in cultured splenocytes, and how exactly it affects MOG_35-55_ or MBP_68-82_-induced neuroinflammation in astrocytes or microglia.

In a previous study, sinomenine, administered to myelin oligodendrocytes glycoprotein_35-55_ (MOG_35-55_)-immunized mice at a dose of 100 mg/kg (i.p., once daily, 18 or 19 days) after the onset of clinical symptoms of multiple sclerosis, was found to attenuate the pathological responses of spinal cord tissue white matter to MOG_35-55_ and the neurological scores associated with the clinical symptoms of multiple sclerosis (Kiasalari et al., 2021) (Table 2). Pre-administration of sinomenine (50, 100, or 200 mg/kg, i.p., once daily for 5 days) to MOG_68-82_-immunized rats also prevented weight loss and delayed disease progression associated with experimental autoimmune encephalomyelitis (EAE) (Zeng et al., 2007). In addition, a novel sinomenine derivative 1032 (15 mg/kg, i.p. treated from day 1 to day 40 after MOG_35-55_ immunization) was shown to reduce EAE scores in mice (Yan et al., 2010) (Table 2). This preventive or ameliorative effect of sinomenine on multiple sclerosis might be related to its anti-inflammatory effect, because sinomenine can suppress MOG_35-55_- or MOG_68-82_-induced infiltration of inflammatory cells and the increase of pro-inflammatory cytokines such as IL-6, IL-1β, IL-18, IL-17A, TNF-α (Zeng et al., 2007; Kiasalari et al., 2021) or interferon-γ (INF-γ), as well as a MOG_35-55_-induced decrease in anti-inflammatory cytokines such as IL-10 in spinal cord tissue (Kiasalari et al., 2021). Administration of sinomenine (100 mg/kg; i.p., once daily, 18 or 19 days; or 50, 100, and 200 mg/kg, i.p., on days 1–3 for five consecutive days) can also suppress the MOG_35-55_-induced increase in NLRP3 and caspase-1 levels and (Kiasalari et al., 2021) or the myelin basic protein_68-82_ (MBP_68-82_)-induced increase in iNOS expression (Gu et al., 2012) in mouse spinal cord tissue (Figure 7; Table 2). These results suggest that suppression of NLRP3-mediated neuroinflammation may mediate the ameliorative effect of sinomenine on disease symptoms in EAE.

The high level of inflammatory responses originates from immune-associated cells including astrocytes. Administration of sinomenine can suppress the MOG_35-55_-induced increase in astrocytes in mouse spinal cord tissue (Kiasalari et al., 2021), suggesting that the astrocyte-mediated neuroinflammatory response may also mediate the amelioration of disease symptoms by sinomenine in animal models of multiple sclerosis. In-depth studies showed that incubation with sinomenine (1 mM) under *in vitro* conditions did not prevent the increase in iNOS expression induced by exogenous IFN-γ and TNF-α in cultured astrocytes, but did prevent the pathological increase in iNOS in primary cultured astrocytes induced by supernatants from cultures of splenocytes treated concomitantly with anti-CD3 antibody and IL-12 (Figure 7) (Gu et al., 2012). Further analysis revealed that IFN-γ might be an important molecule mediating the above process, because incubation with sinomenine can suppress the expression of IFN-γ and its upstream regulator T-bet, which is induced by myelin basic protein_68-82_ (MBP_68-82_) in both rat spinal cord tissue and cultured splenocytes (Figure 7) (Gu et al., 2012). Therefore, the anti-iNOS effect of sinomenine in astrocytes in multiple sclerosis might be due to the suppression of the T-bet-IFN-γ signaling in splenocytes. However, this is only a hypothesis. It should be further investigated in future studies.

### Pharmacological effects of sinomenine in morphine dependence

Morphine dependence is a well-known public problem for which there are no effective treatments, with the exception of agonist substitution therapy, which uses long-acting opioid agonists. However, this method can cause a variety of problems, including relapse and triggering negative feelings, such as anxiety and anhedonia (Veilleux et al., 2010; Li et al., 2017). Therefore, there is an urgent need to search for new drugs or strategies to treat morphine dependence. In a study by Fang et al. (2017), it was found that administration of sinomenine (on days 5–7 after the preconditioning phase and the first and second sessions on day 4; 80 mg/kg, i.p.) prevented the morphine-induced increase in the time mice spent in the non-preferred white compartment in the conditioned place preference test, suggesting that sinomenine could be a potential drug for treating morphine dependence.

**FIGURE 8:**
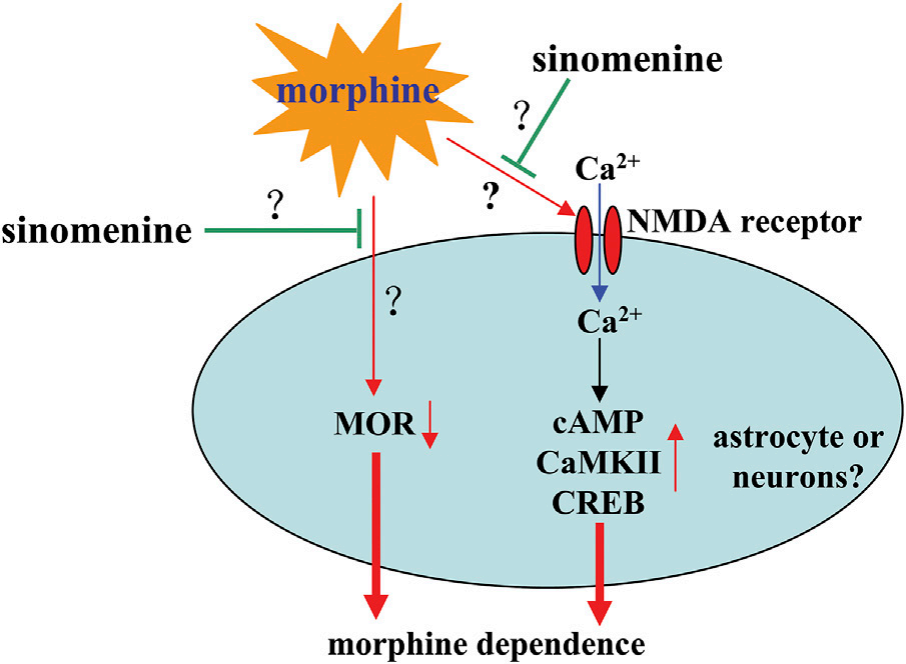
Effects and mechanisms of sinomenine in morphine dependence. Sinomenine administration was found to suppress the morphine-induced increase in Ca^2+^-cAMP-CaMKII-CREB signaling, which likely contributes to the amelioration of morphine dependence Ou et al. (2018). Administration of sinomenine may also suppress morphine-induced down-regulation of MOR in the brains of mice Fang et al. (2017), the function of which is currently unclear. In addition, researches do not yet know how sinomenine inhibits morphine-induced activation of NMDAR signaling.

Previous studies have shown that dopamine-producing TH-positive neurons (Yamamoto et al., 2010) and a subunit of the N-methyl-D-aspartate receptor (NMDAR), NR2B, play an important role in conditioned place preference (Zhou et al., 2010). Sinomenine administration can prevent the morphine-induced increase in the expression of TH and NR2B, suggesting that sinomenine appropriately inhibits morphine dependence by enhancing the signaling function mediated by TH and NR2B. This hypothesis is somewhat supported by the finding that incubation with sinomenine decreases intracellular Ca^2+^, cAMP, phospho-CaMKII, or phospho-CREB in morphine-treated SH-SY5Y cells and in the hippocampus of morphine-stimulated mice (Ou et al., 2018) (Figure 8; Table 2). Considering that morphine addiction is an aberrant deviant form of learning and memory (Chen et al., 2022b) and that pathological changes in NMDAR, CaMKII, or CREB are known to correlate with learning and memory (Ko et al., 2018), these results suggest that sinomenine may attenuate morphine dependence by reducing NMDAR-mediated Ca^2+^ influx and thus cAMP-CaMKII-CREB signaling (Figure 8). However, this is currently only a hypothesis. Further studies should be conducted to investigate the relationship between NMDAR-Ca^2+^-CaMKII-CREB signaling and the effect of sinomenine against morphine dependence.

The mu opioid receptor (MOR) and the delta opioid receptor (DOR) are two important receptors that mediate the rewarding effects of opioid systems and elicit conditioned place preference behavior in animals (Sante et al., 2000). Expression of MOR, but not DOR, has been shown to be reduced in the hippocampus of mice during morphine exposure, and treatment with sinomenine can significantly reverse this reduction (Fang et al., 2017) (Figure 8; Table 2). In addition, administration of sinomenine (80 mg/kg) attenuated the morphine-induced decrease in opioid receptors including MOR and DOR in the brains of morphine-exposed zebrafish (Lin et al., 2021). These results suggest that enhancement of MOR function may mediate the effect of sinomenine against morphine dependence. This hypothesis may be consistent with a previous finding that sinomenine has the ability to activate MOR and delay analgesic tolerance to morphine in mice (Wang et al., 2008). However, there is a confusing question that should be answered in this section: it has been reported that cAMP-CREB signaling enhances the gene expression of MOR (Lee and Lee, 2003), but under the condition of morphine treatment, the increased cAMP-CREB signaling was not accompanied by an increase in MOR, but by a decrease in MOR. In future studies, researchers should further clarify why morphine treatment reduces the expression of MOR in mouse brain and why sinomenine can reverse this effect. In addition, researchers should clarify why there is a differential effect of sinomenine on the expression of MOR and DOR in morphine-stimulated mice and zebrafish.

Astrocytes have been shown to play an important role in morphine dependence (Darvishmolla et al., 2022; Murlanova et al., 2022). Previous studies have shown that administration of sinomenine to mice (60 mg/kg, i.p., 45 min before morphine injection, 3 days) or incubation with sinomenine in SH-SY5Y cells (100 µM, 12 h) inhibits morphine-induced activation of astrocytes and, in particular, induces a modification of astrocyte exosomes that has potentially neuroprotective effects (Ou et al., 2018) (Table 2). This view is supported by the finding that treatment of SH-SY5Y cells with exosomes derived from astrocytes stimulated by sinomenine reduced the morphine-induced increase in cAMP, Ca^2+^, and phospho-CaMKII and phospho-CREB in SH-SY5Y cells (Ou et al., 2018) (Table 2). Although this result is from neuronal cell lines, researchers might hypothesize that the effect of sinomenine on NMDAR and the subsequent inhibition of intracellular Ca^2+^-cAMP-CaMKII-CREB signaling may be due to the effect of sinomenine on astrocytes (Figure 8). However, this is only a hypothesis. It should be investigated in future studies.

Studies in zebrafish suggest that the effect of sinomenine on morphine dependence may depend on the gut microbiota. First, during sinomenine (80 mg/kg) can reverse the morphine-induced increase of Fusobacteria and decrease of Actinobacteria in the gut microbiota of zebrafish when reversing the morphine-induced dependence behavior, (Chen et al., 2020) (Table 2). Second, administration of sinomenine can reverse the decrease in tight junction proteins and opioid receptor mu 1 (OPRM1) and opioid receptor delta 1 (OPRD1) in the morphine-stimulated brain and intestine of zebrafish (Chen et al., 2020) (Table 2). Third, sinomenine administration was found to reverse the morphine-induced increase in the expression of DRD2A, 5-hydroxytryptamine 2A (HTR2A), BDNF, and neurotrophic tyrosine kinase receptor type 2 (NTRK2) in zebrafish brain and gut (Chen et al., 2020) (Table 2). Interestingly, all of these effects were reversed by antibiotic treatment (Chen et al., 2020), suggesting that the gut microbiota is required for proper regulation of morphine dependence by sinomenine. Recently, changes in the homeostasis of the gut microbiota may play a crucial role in the development of morphine dependence (Lee et al., 2018; Chen et al., 2021). Therefore, it is necessary to thoroughly investigate the role of gut microbiota in the regulation of morphine dependence by sinomenine and even some other similar small compounds.

### Pharmacological effects of sinomenine in glioma

Glioblastoma is a common cancer in humans that can be treated with a variety of methods including radiation therapy, chemotherapy, and surgery (Janjua et al., 2021). However, it has been shown that the median survival time of glioma patients treated with current therapies is less than 15 months (Yuan et al., 2022). Therefore, the search for new drugs to treat glioma is an urgent goal for scientists and clinicians. *In vitro* studies have shown that incubation with sinomenine (0.0625, 0.125, 0.25, 0.5, and/or 1 mM, for 24, 48, or 72 h) can inhibit the cell viability of U87 and SF767 cells, two human glioblastoma cell lines (Jiang et al., 2018). This effect of sinomenine was not related to caspase-dependent cell death, as there was no significant increase in the expression of cleaved caspase-3 in these cells (Jiang et al., 2018) (Table 3). Alternatively, researchers found that sinomenine induces the accumulation of LC3B-II and increases the expression of lysosomal-associated membrane protein-1 (lamp-1) and cathepsin B/D in U87 and SF767 cells by promoting nuclear translocation of transcription factor EB (TFEB) (Tan et al., 2022) (Table 3), suggesting that sinomenine reduces cell viability by inducing autophagy. This view can be supported by the finding that pre-treatment with autophagy inhibitors increased the viability of U87 and SF767 cells (Jiang et al., 2018). Further analysis showed that the antioxidant N-acetylcysteine, Akt-specific activator, and c-Jun N-terminal kinase (JNK)-specific inhibitor attenuated sinomenine-triggered autophagy in U87 and SF767 cells (Jiang et al., 2018), suggesting that the ROS-Akt-JNK signaling may mediate the autophagy-inducing effect of sinomenine. In addition to promoting cell death, incubation with sinomenine (0.125, 0.25, or 0.5 mM, 24 h) also promoted G0/G1 arrest and inhibited migration and invasion of U87 and SF767 cells, which may be mediated by suppression of NF-κB activation and MMP-2/9 expression and reversal of NF-κB-mediated epithelial-mesenchymal transition (EMT) (Jiang et al., 2017) (Table 3).

**TABLE 3:** Comprehensive information about the pharmacological effects and mechanisms of sinomenine in glioma.

Pharmacological effect	Object	Drug administration	Possible mechanisms	References
Inhibit cell viability	*U87 cells *SF767 cells	0.0625, 0.125, 0.25, 0.5, and/or 1 mM, for 24, 48, or 72 h	Trigger autophagy *via* ROS-Akt-JNK signaling	Jiang et al. (2018)
*Promote G0/G1 arrest	*U87 cells *SF767 cells	0.125, 0.25, or 0.5 mM, 24 h	*Suppress NF-κB activation and MMP-2/9 expression *reverse NF-κB-mediated epithelial-mesenchymal transition	Jiang et al. (2017)
*Inhibit migration and invasion				
*Reduce tumor volume/weight	mice	*75 or 150 mg/kg	Trigger autophagy	Jiang et al. (2017)
*Inhibit cell proliferation		*i.p.		
*Induce cell cycle arrest		*Once daily, 14 days		

Based on the above results, the investigators found that administration of sinomenine at a dose of 75 or 150 mg/kg (i.p., once daily, 14 days) in nude mice transplanted with U87 cells reduced tumor volume and weight, inhibited cell proliferation, and induced cell cycle arrest by increasing LC3B and cathepsin B/D expression, down-regulating p62 expression, and reducing the expression of proteins related to cell proliferation and migration (Jiang et al., 2017) (Table 3). Taken together, these results show that sinomenine can also induce autophagy under *in vivo* conditions and exert a significant anti-proliferation effect on glioma cells in animal experiments. Therefore, sinomenine may be a valuable agent for the development of new strategies against glioma.

## Conclusion

In this review, the authors outline the pharmacological effects of sinomenine on CNS disorders other than pain in preclinical *in vitro* and *in vivo* models. Sinomenine may exert preventive and/or ameliorative effects on CNS disorders by inhibiting neuroinflammation and oxidative stress, which are linked *via* NO and ROS (Yang et al., 2016a; Fu et al., 2018; Gao et al., 2018; Ramazi et al., 2020; Sharma et al., 2020; Kiasalari et al., 2021; Bao et al., 2022; Rostami et al., 2022). The changes in molecules such as IκB-α (Qin et al., 2018), NF-κB (Jiang et al., 2017; Qin et al., 2018), JNK (Jiang et al., 2018), Nrf2 (Bi et al., 2021), Akt (Bao et al., 2022), AMPK (Qiu et al., 2016a), CREB (Li et al., 2018), and BDNF (Li et al., 2018) may mediate the effects of sinomenine in CNS disorders, including cerebral ischemia, intracerebral hemorrhage, TBI, AD, PD, epilepsy, sleep disturbance, depression, multiple sclerosis, and morphine tolerance. Regulation of NF-κB and Nrf2 by sinomenine may be the most common. In glioma, sinomenine treatment was found to suppress tumor cell proliferation and migration by inducing autophagy-mediated cell death (Jiang et al., 2017; Jiang et al., 2018). The fact that sinomenine targets DRD2 (Qiu et al., 2016b), NMDAR (Fang et al., 2017), and GABA_A_ receptors (Yoo et al., 2017) may be of further interest to researchers to explore the profound mechanisms that regulate CNS disorders, especially those associated with changes in neuronal activity.

Overall, as a drug that can be delivered into the brain (Liu et al., 2005; Long et al., 2010), sinomenine may have the potential to be developed as a drug to prevent or treat a variety of CNS disorders. However, it should be noted that although sinomenine can enter the brain (Liu et al., 2005; Long et al., 2010; Gao et al., 2019a), its clinical application and development are largely limited by characteristics such as weak efficacy, short biological half-life, and potential side effects at high doses (Fu et al., 1963; Yamasaki, 1976; Gao et al., 2019a). Therefore, the development of novel strategies to improve bioavailability could promote the use of sinomenine for the prevention or treatment of CNS disorders. Indeed, a number of experiments have been launched in recent years to provide indirect evidence for this goal. For example, the novel sinomenine derivative designated 1032, synthesized by embedding a small nitrogen-containing heterocycle into the vicinal dicarbonyl functionality of the C-ring of sinomenine, has been shown to exhibit enhanced immunosuppressive activity in immune cells (Yan et al., 2010). In addition, a drug form of sinomenine conjugated to a hydroxyl-terminated generation 4 poly dendrimer called D-Sino was found to increase the intracellular availability of sinomenine in immune cells, thereby enhancing the anti-inflammatory effect of sinomenine in an animal model of TBI (Sharma et al., 2020). Since sinomenine has been used in several clinical trials for the treatment of rheumatoid arthritis (Liu et al., 2016; Huang et al., 2019), further exploration of strategies to improve the efficacy of sinomenine when ingested may open new avenues for the development of sinomenine as a novel and clinically available drug for the prevention or treatment of CNS disorders and rheumatoid arthritis.
